# Prognostic impact of PD-L1 and TIGIT expression in non-small cell lung cancer following concurrent chemo-radiotherapy

**DOI:** 10.1038/s41598-023-29724-4

**Published:** 2023-02-25

**Authors:** Masataka Mori, Masatoshi Kanayama, Taiji Kuwata, Takehiko Manabe, Yukiko Nemoto, Natsumasa Nishizawa, Rintaro Oyama, Hiroki Matsumiya, Yusuke Nabe, Akihiro Taira, Masaru Takenaka, Kazue Yoneda, Koji Kuroda, Fumihiro Tanaka

**Affiliations:** Second Department of Surgery, University of Occupational and Environmental Health, 1-1 Iseigaoka, Yahatanishi-Ku, Kitakyushu, 807-8555 Japan

**Keywords:** Cancer microenvironment, Lung cancer

## Abstract

We investigated the effect of preoperative therapy for non-small cell lung cancer on programmed death-ligand 1 (PD-L1), programmed death-1 (PD-1), poliovirus receptor (CD155), and T cell immunoglobulin and immunoreceptor tyrosine-based inhibitory motif (ITIM) domain (TIGIT) expression and prognosis with the cases of 28 patients received preoperative concurrent chemo-radiotherapy (cCRT) and 27 received preoperative drug therapy. The post-treatment PD-L1 expression was higher in cCRT group than in the drug therapy (50.0% vs 5.0%, *p* = 0.000), whereas that of CD155 did not significantly differ (40.0% vs 60.0%, *p* = 0.131). The PD-1 expression was not significantly different between the cCRT and drug therapy groups (51.1% vs 42.9%, *p* = 0.076), while the TIGIT was significantly higher in the cCRT group (41.5% vs 34.0%, *p* = 0.008). The patients who received cCRT resulted in elevated PD-L1and TIGIT values had a worse prognosis (*p* = 0.008). The PD-L1 and TIGIT expression after cCRT was significantly higher than after drug treatment. The cCRT population with high expression of both had a significantly poorer prognosis, indicating elevation of PD-L1 and TIGIT after cCRT as a negative prognostic factor. Combination therapy with anti-PD-L1 and anti-TIGIT antibodies after cCRT may contribute to an improved prognosis.

## Introduction

Non-small cell lung cancer (NSCLC) accounts for the majority of lung cancer, which is globally the foremost cause of cancer death^1^. Many newly diagnosed NSCLC patients are with metastatic lesions and traditionally have been treated first with a platinum doublet chemotherapy with limited efficacy. However, the improved prognosis noted with immunotherapies such as programmed death-1/programmed death-ligand 1 (PD-1/PD-L1) axis immune checkpoint inhibitors (ICIs) has changed the treatment strategy^2^. Despite their durable response, ICI monotherapy has a response rate of at most 20%, leading to the development of combinations of ICIs. Basic research reported good prospects for combination therapy with multiple ICIs^3,4^, which has also been proven in clinical studies^5–8^. In the phase II CITYSCAPE trial, anti-T cell immunoglobulin and immunoreceptor tyrosine-based inhibitory motif (ITIM) domain (TIGIT) antibody tiragolumab combined with the PD-L1 inhibitor atezolizumab significantly improved the overall response rate compared to anti-PD-L1 monotherapy (37% vs. 21%) (37% vs. 21%)^9^. In patients with tumors with high PD-L1 expression (n = 58), the objective response rate was 69.0% (95% CI 50.4–87.5) verus 24.1% (6.8–41.4) and median progression-free survival was 16.6 (95% CI 5.5–22.3) vs 4.1 months [2.1–6.8; HR 0.29 (95% CI 0.15–0.053)]^10,11^. However, the phase 3 SKYSCRAPER-01 study did not meet its co-primary endpoint of progression-free survival^12^.

Approximately one-third of newly diagnosed NSCLC patients is locally advanced disease irrelevant to surgical resection^13^. Concurrent chemo-radiotherapy (cCRT) has been a typical treatment for these patients despite 5-years overall survival (OS) rates of as low as 15%^14^. Recently, the PACIFIC, a phase III randomized, double-blind trial of durvalumab (an antibody against programmed death ligand-1: PD-L1) vs. placebo, demonstrated that the consolidation treatment with durvalumab for patients without disease progression after cCRT significantly improved their prognosis (4-years OS; 49.6% vs. 36.3%)^15–18^. As regards an association between the promising combination, anti-TIGIT and anti-PD-L1 therapy, and radiotherapy (RT), RT-induced TIGIT and PD-L1 upregulation and RT in combination with anti-TIGIT and PD-L1 inhibitor showed a complete response (CR) rate of 90% in a mouse model^19^. RT combined with anti-TIGIT and anti-PD-L1 antibodies is considered to be a hopeful approach. To date, however, no clinical evidence has been reported as concerns the association between RT and alteration in the immune-marker state, TIGIT and PD-L1 expression, and the rationale of RT with anti-TIGIT and anti-PD-L1 antibody drugs.

Previously, we reported a significant up-regulation of PD-L1 expression on the tumor cells and an increased stromal density of CD8 + tumor infiltrative lymphocytes (TILs) in paired clinical samples before and after cCRT^20^. In this study, we analyzed 14 more cases in addition to the 41 cases reported in the previous study. Here, we aimed to assess in the same and subsequent clinical samples the effect of RT on PD-L1 and TIGIT expression together with their major counterparts, PD-1 and poliovirus receptor (CD155), and their prognostic impact. We compared the expression and influence of the markers on prognosis in patients undergoing either cCRT or drug therapy before surgery.

## Results

### Characteristics of patients

Characteristics of the patients and their treatments are summarized in Table 1. We compared 28 patients who underwent cCRT with 27 patients who underwent drug therapy before surgery. All patients of the cCRT group received platinum doublet chemotherapy and radiotherapy concurrently with a median dose of 60 Gy. In the drug therapy group, there were TKI and bronchial artery infusion with cisplatin, as well as platinum doublets.

**Table 1:** Characteristics of patients classified by induction therapies.

	cCRT group	Drug-therapy group	*p*-value
	(n = 28)	(n = 27)	
Sex			0.931
Male	20 (71.4%)	19 (70.4%)	
Female	8 (28.6%)	8 (29.6%)	
Age, median (range)	66 (40–79)	69 (46–84)	0.111
Smoking status			0.937
Never	7 (25.0%)	7 (25.9%)	
Former or current	21 (75.0%)	20 (74.1%)	
Brinkman index, median (range)	765 (0–1760)	940 (0–1880)	0.734
Comorbidity	20 (71.4%)	24 (88.9%)	0.106
Histology			0.993
Adenocarcinoma	16 (57.1%)	15 (55.6%)	
Squamous	10 (35.7%)	10 (37.0%)	
Others	2 (7.1%)	2 (7.4%)	
Clinical stage			0.729
IIA or IIB	6 (21.4%)	4 (14.8%)	
≥ IIIA	22 (78.6%)	23 (85.2%)	
Drug therapy			0.001
Platina doublet	28 (100%)	15 (55.6%)	
Other cytotoxic	0 (0%)	1 (3.7%)	
Bronchial artery infusion	0 (0%)	4 (14.8%)	
Tyrosine kinase inhibitor	0 (0%)	7 (25.9%)	
Radiotherapy dose (Gy), median (range)	60 (44–70)	–	0.000
Clinical response			0.587
PD or SD	10 (35.7%)	12 (44.4%)	
PR	18 (64.3%)	15 (55.6%)	
Resection			0.469
Lobectomy	25 (89.3%)	22 (81.5%)	
Pneumonectomy	3 (10.7%)	5 (18.5%)	
Effect of induction therapy			0.000
Ef1	7 (25.0%)	21 (77.8%)	
Ef2	21 (75.0%)	6 (22.2%)	

**Figure 1:**
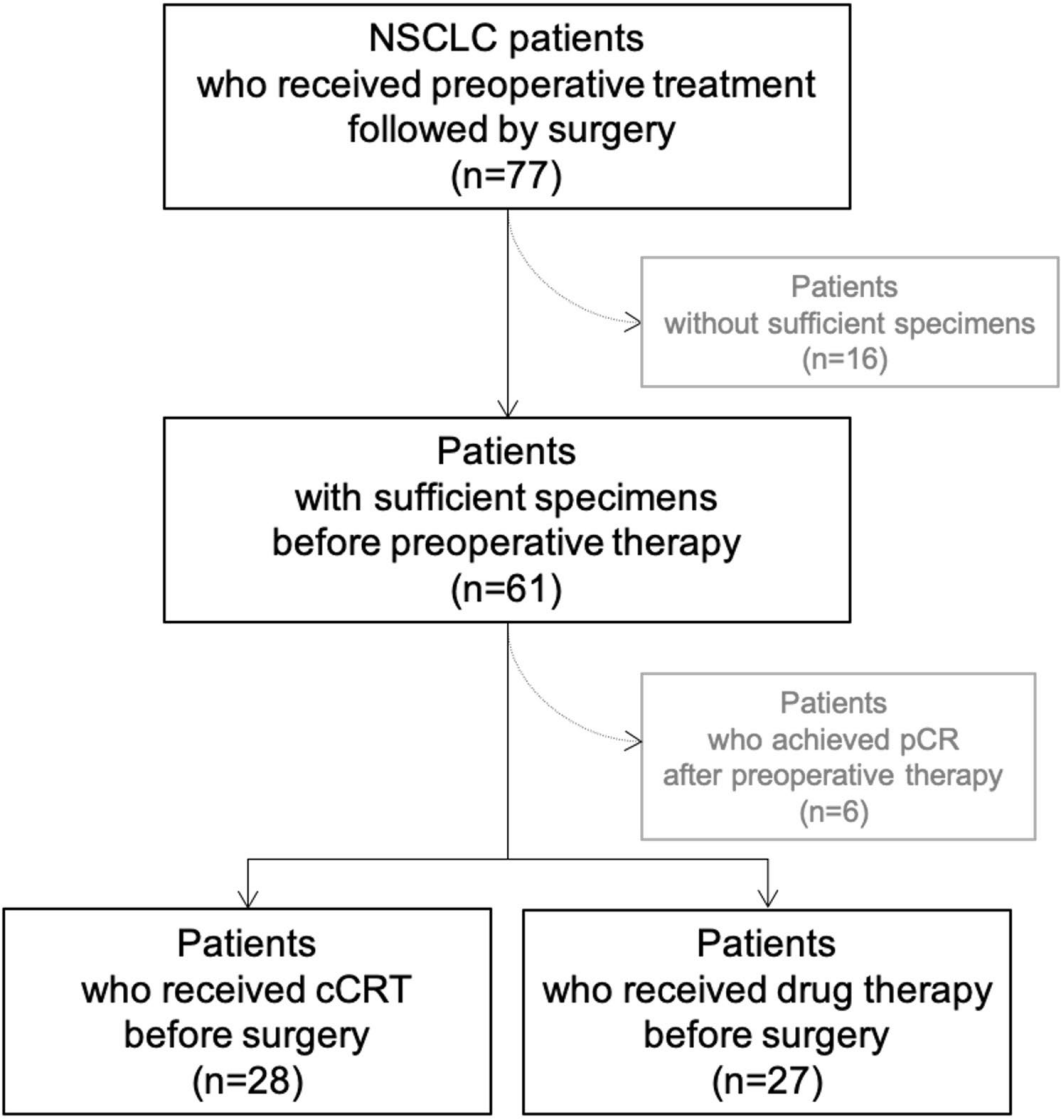
Flow diagram of the present study participants.

The therapeutic effect of the cCRT was higher than in the drug therapy group. The proportion of Ef2 was 75.0% in the cCRT group vs 22.2% in the drug therapy group (*p* = 0.000) (Fig. 1).

### PD-L1 and CD155 expression

**Figure 2:**
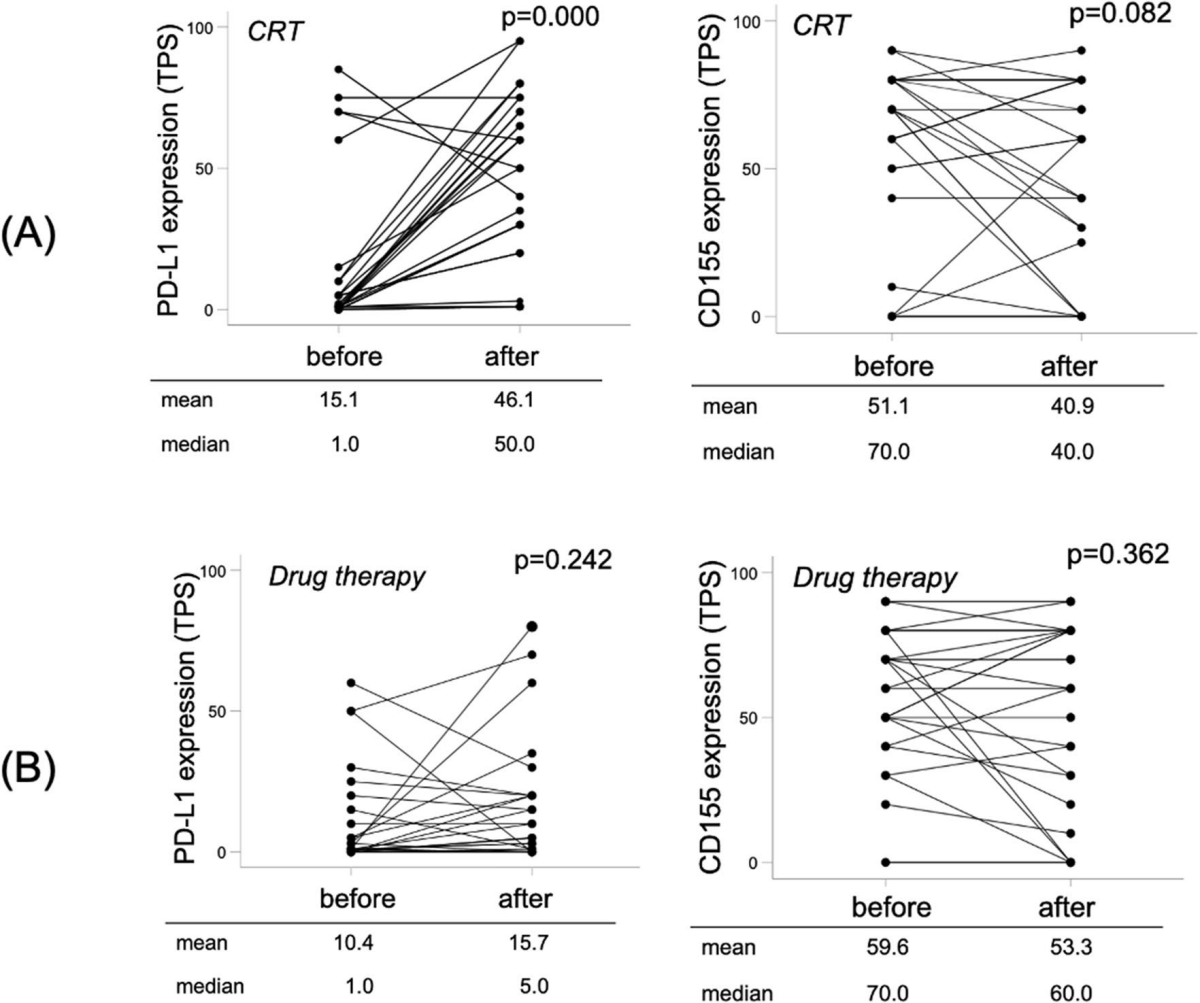
Alteration in PD-L1 and CD155 expression on tumor cells. The results are shown according to (**A**) the patients treated with concurrent chemo-radiotherapy and (**B**) those treated with drug therapy.

To assess the effect of induction therapy on PD-L1 and CD155 expression of the tumor, we evaluated their expression before and after induction therapy. Whereas cCRT group demonstrated a statistically significant upregulation in PD-L1 (*p* = 0.000) and a downregulated trend in CD155 expression (*p* = 0.082, Fig. 2A), the drug therapy group showed no significant difference in PD-L1 (*p* = 0.242) and CD155 expression (*p* = 0.362, Fig. 2B). The post-treatment median TPS of PD-L1 was tenfold higher in the cCRT group than in the drug therapy (50.0% vs 5.0%, *p* = 0.000). The post-treatment median TPS of CD155 was not significantly different between the two groups (40.0% vs 60.0%, *p* = 0.131).

### PD-1 and TIGIT expression of CD8 + lymphocytes

**Figure 3:**
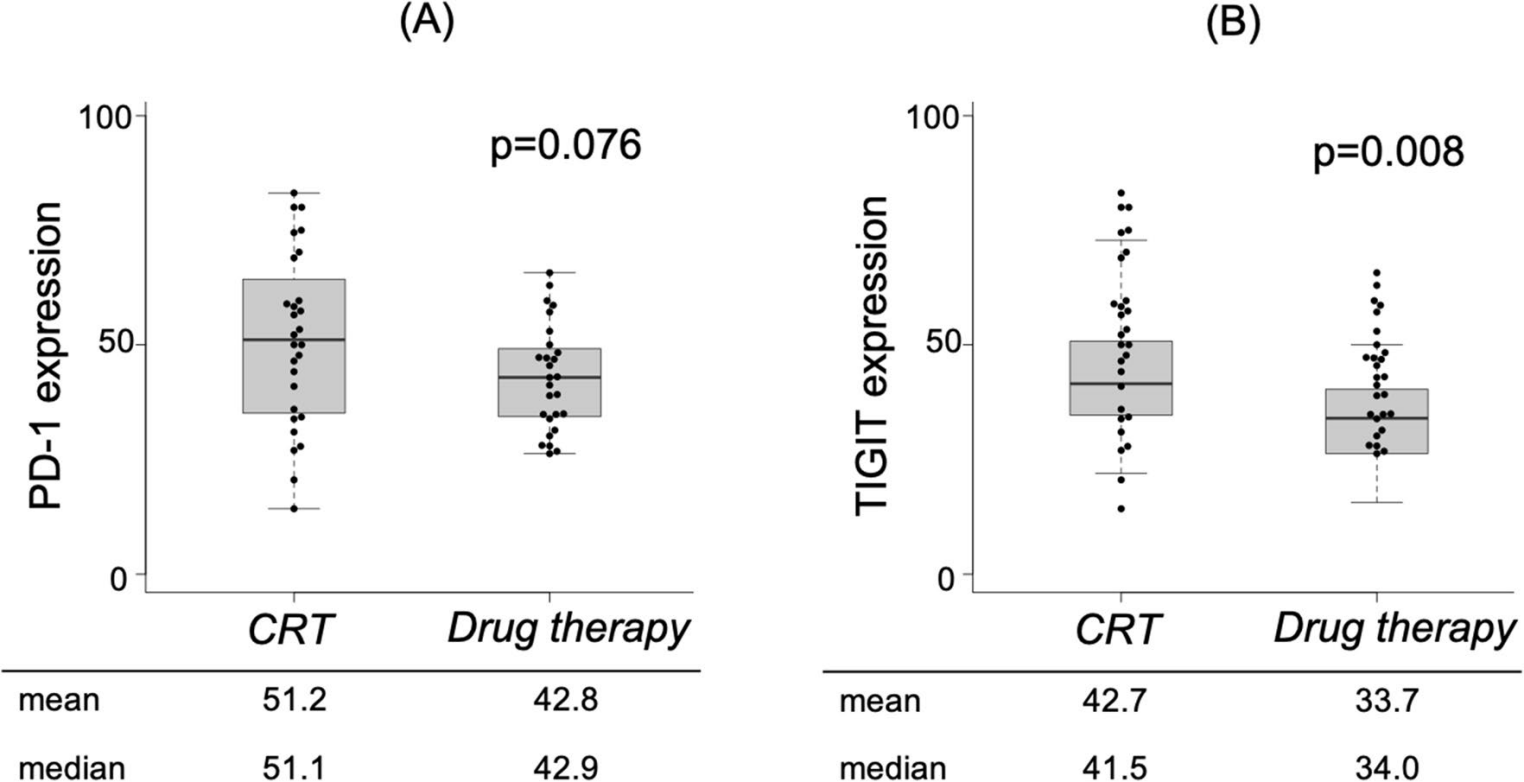
Expression rate of the TIL markers on CD8 + cells after induction therapy. The results are shown according to (**A**) PD-1 and (**B**) TIGIT expression.

To evaluate the influence of induction therapy on PD-1 and TIGIT expression of CD8 + lymphocytes, we assessed their expression rate in the post-treatment specimens. The PD-1 expression was not statistically different between the cCRT and drug therapy group (51.1% vs 42.9%, *p* = 0.076, Fig. 3A). The TIGIT expression was statistically higher in the cCRT group compared to the drug therapy group (41.5% vs 34.0%, *p* = 0.008, Fig. 3B).

### Cut-off value for prognostic analyses

A receiver operating characteristic curve analysis of PD-L1, CD155, PD-1, and TIGIT for prediction of death was performed, indicating an optimal cut-off value of 25% (AUC = 0.640), 45% (AUC = 0.425), 40% (AUC = 0.483), and 40% (AUC = 0.477), respectively. Based on these results, each case was classified as marker-high (cut-off value or higher) or marker-low (less than the cut-off value).

### Overall Survival regarding each biomarker

**Figure 4:**
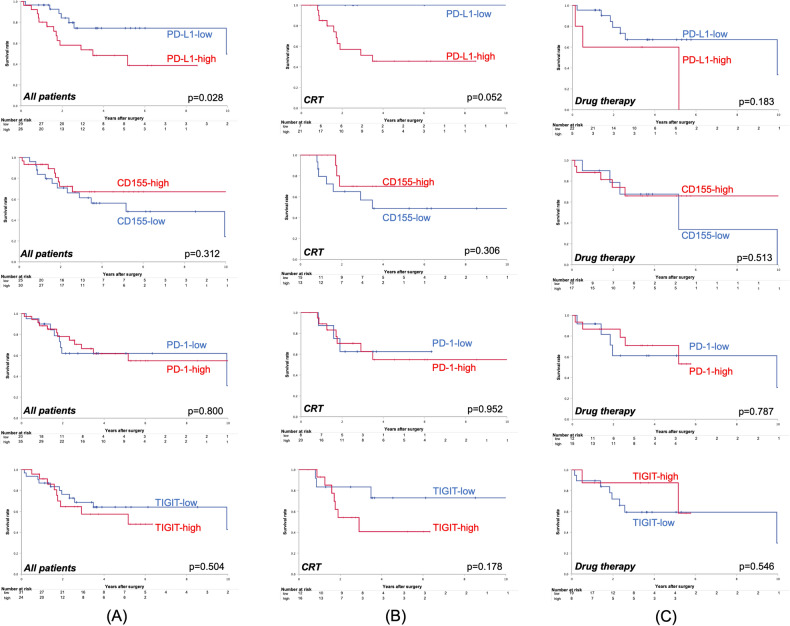
Overall survival curves compared with the expression of immune markers, PD-L1, CD155, PD-1, and TIGIT. The results are shown according to (**A**) all patients, (**B**) patients treated with cCRT, and (**C**) those treated with drug therapy.

The OS curves in respect to PD-L1, CD155, PD-1, and TIGIT expression of all patients are shown in Fig. 4A. Except for PD-L1 expression, there was no significant correlation between CD155, PD-1 and TIGIT expression and OS (PD-L1: *p* = 0.028, CD155: *p* = 0.312, PD-1: *p* = 0.800, TIGIT: *p* = 0.504). The OS curves for each marker of the cCRT group are demonstrated in Fig. 4B. There was no significant relation between any marker and OS (PD-L1: p = 0.052, CD155: *p* = 0.306, PD-1: *p* = 0.952, TIGIT: *p* = 0.178). The OS curves regarding each marker of the drug therapy group are presented in Fig. 4C. There was no significant correlation between each marker and OS (PD-L1: *p* = 0.183, CD155: *p* = 0.513, PD-1: *p* = 0.787, TIGIT: *p* = 0.546).

### Overall survival regarding PD-L1-high and TIGIT-high

**Figure 5:**
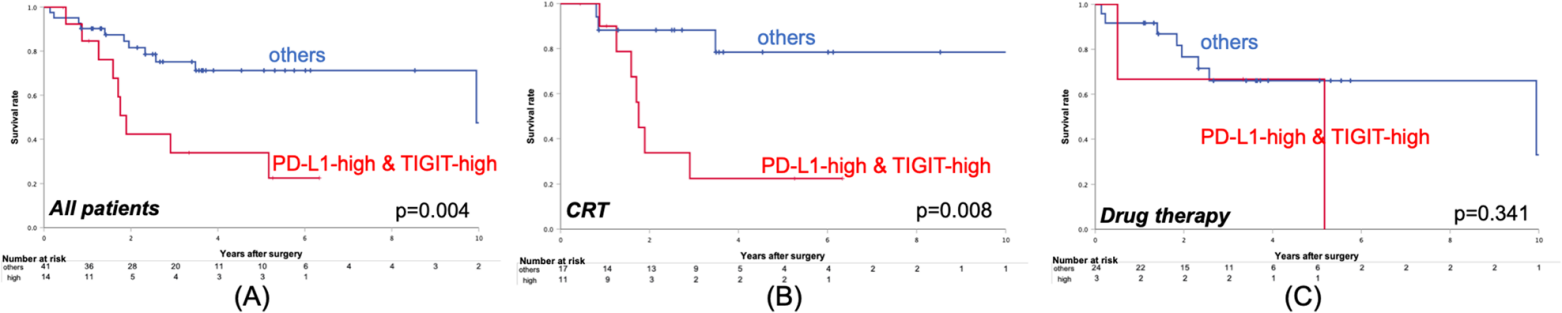
Overall survival curves regarding the expression of PD-L1 together with TIGIT. The results are shown according to (**A**) all patients, (**B**) patients treated with cCRT, and (**C**) those treated with drug therapy.

Figure 5 shows the OS in PD-L1-high and TIGIT-high patients. The OS of the patients with elevated PD-L1 and TIGIT was significantly worse (Fig. 5A, *p* = 0.004). In the CRT group, the survival in cases with elevated PD-L1 and TIGIT was significantly worse (Fig. 5B, *p* = 0.008), whereas there was no difference in the drug therapy group (Fig. 5C, *p* = 0.341).

## Discussion

In this retrospective study, we evaluated the expression of immune markers, PD-L1, CD155, PD-1, and TIGIT after cCRT or drug therapy and their relation to the prognosis in a series of patients with NSCLC. Our results demonstrated that the expression of PD-L1 was upregulated and the expression rate of TIGIT was high after cCRT relative to that after drug therapy, and the patients with NSCLC after cCRT with high expression of both PD-L1 and TIGIT had a significantly poor prognosis. These findings suggest that combination therapy with anti-PD-L1 and anti-TIGIT antibodies after cCRT may contribute to an improved prognosis.

We found that the samples after cCRT demonstrated a significant increase in PD-L1 expression (*p* = 0.000) in comparison to before the treatment. This result was consistent with our previous study^20^ and is supported by several preclinical and clinical studies^21–23^. Regarding CD155, our result showed that the samples after cCRT tend to be a lower expression (*p* = 0.082) compared to before the therapy. The impact of RT on CD155 expression has not been reported so far. The PD-1 expression rate of CD8 + cells was prone to be high in the cCRT group relative to in the drug therapy group, though the difference was not statistically significant (*p* = 0.076). While some experiments with mouse models suggested that RT had an effect of increasing PD-1 expression^23–26^, another study showed no significant difference in PD-1^21^. In our study, the TIGIT expression rate was higher in the cCRT group than in the drug therapy group (*p* = 0.008). A preclinical study revealed that RT leads to increased TIGIT expression^19^. Our study may indicate that RT for NSCLC is associated with upregulation of PD-L1, downregulation of CD155, and high expression of PD-1 and TIGIT.

Next, we focused on the prognostic impact of the immune markers. Of the four markers, PD-L1 was the only statistically significant prognostic factor for OS, with a poor prognosis for high PD-L1 expression (Fig. 4A). This trend was prominent in the CRT group and was not observed in the drug therapy group.

In NSCLC, reports of PD-L1 as a prognostic factor have been inconclusive^27–33^. This study differs from other studies in that all the patients had undergone the preoperative treatments. Tumors that can express PD-L1 due to the high density of CD8 + cells after cCRT and their IFN-γsecretion^34,35^ may be associated with poor prognosis through their ability to escape the immune system. Furthermore, in this study, the prognosis was significantly poor in the subgroup with high expression group of both PD-L1 and TIGIT, and this event was also remarkable in the cCRT group and was not observed in the drug therapy group (Fig. 5). In this subgroup, exhaustion of T cells and NK cells by TIGIT^36^, in addition to immune escape through PD-L1, may have resulted in tumor progression and a worse prognosis. These results indicate that the combined use of anti-PD-L1 antibody and anti-TIGIT antibody after cCRT for NSCLC may improve their prognosis.

Several limitations of our study warrant mention. First, the study was conducted under a retrospective design at a single center. Although we decided upon an ideal cut-off value for each marker using ROC analysis, this should be confirmed in a larger cohort. Second, the patients in this study had a wide variety of backgrounds. The RT doses ranged widely and the kinds of the drug therapy were diverse and included TKIs. Third, it would be desirable to have a baseline assessment in evaluating the impact of preoperative treatment, which is not being done. Finally, the present study was conducted on a small number of subjects. These might affect the results. Allowing for these issues, however, we believe that our findings are valuable in this age of ICIs combination therapy considering the scarcity of specimens after preoperative therapy.

## Conclusion

PD-L1 expression after cCRT was higher than before the therapy and TIGIT expression rate was higher in the cCRT group than in the drug therapy group. The prognosis of patients with elevation of both PD-L1- and TIGIT patients undergoing cCRT was poorer than that of the others in the cCRT group. These findings may indicate a clinical rationale for combining anti-PD-L1 and anti-TIGIT antibodies after cCRT.

## Materials and methods

### Patients

In this retrospective study, we reviewed cases of NSCLC patients who had received preoperative therapy followed by surgery from April 2006 to October 2020 in our department (Fig. 1). Inclusion criteria were available tumor specimens before and after preoperative therapy satisfactory for pathological evaluation of PD-L1 and CD155, and surgical specimens satisfactory for assessment of PD-1 and TIGIT expression of stromal CD8 + TIL. Of 77 patients who had undergone preoperative therapy. Sixteen cases were excluded because of insufficient formalin-fixed paraffin-embedded samples before therapy, and six were excluded because of a lack of viable tumor cells in surgical specimens due to pathological CR. We reviewed the remaining 55 patients, of whom 28 underwent preoperative cCRT and 27 underwent preoperative drug therapy alone.

The study protocol was approved by the University of Occupational and Environmental Health, Japan (UOEHCRB21-154), and the study was performed in accordance with the Declaration of Helsinki. Informed consent was obtained from all subjects or their legal guardians.

### Pathological effect of induction therapy

The effect of induction therapy was assessed based on the criteria in the classification of lung cancer by the Japan lung cancer society. Ef3 means that there are no viable tumor cells. Ef2 means that less than one-third of tumor cells were viable. Ef1 means that more than one-third of tumor cells were viable despite the existence of a therapeutic effect.

### Evaluation of tumor immune microenvironment

Consecutive 4-μm tissue sections were used for hematoxylin–eosin staining and immunohistochemistry (IHC) staining. The objects of IHC in this study were PD-L1, CD155, PD-1 of CD8 + lymphocytes, and TIGIT of CD8 + lymphocytes. PD-L1 staining was performed as described previously^37^. Tissue sections were deparaffinized and incubated in ethylenediaminetetraacetic acid (pH 8.0) at 98 °C for antigen retrieval. Endogenous peroxidase was blocked using 3% H2O2. After nonspecific reaction blocking with Protein Block Serum-Free (Agilent Technologies, Palo Alto CA, USA), we incubated sections with rabbit anti–PD-L1 monoclonal antibody (clone E1L3N, Cell Signaling Technology Japan, Tokyo, Japan) diluted at 1:200 and washed and incubated with a detection reagent (SignalStain Boost IHC Detection Reagent HRP Rabbit, Cell Signaling Technology, Tokyo, Japan). The sections were visualized with DAB + Liquid (Agilent Technologies) and counterstained with Mayer’s hematoxylin. Retrieval of primary antibody and secondary antibody of the remaining IHC were as follows: as described previously^5^, IHC of CD155 was performed with citrate buffer (pH 6.0) at 98 °C for antigen retrieval and mouse anti-CD155 monoclonal antibody (clone B6, Santa Cruz Biotechnology, Texas, US) and Histofine Simple Stain, MAX-PO (Nichirei Bioscience, Tokyo, Japan). The other than these the same as described above. For CD8 and PD-1 multistaining, we used citrate buffer (pH 6.0) at 98 °C as antigen retrieval buffer and rabbit anti-CD8 antibody (clone 1779R, Novus Biologicals, Centennial CO, US) diluted at 1:300 and mouse anti-PD-1 antibody (clone NAT105, Abcam, Cambridge, UK) diluted at 1:50 as the primary antibody and Alexa Flour 488-conjugated goat anti-rabbit IgG (Thermo Fisher Scientific, Massachusetts, US) diluted at 1:500 and Alexa Fluor 594-conjugated goat anti-mouse IgG (Thermo Fisher Scientific, Waltham MA, US) diluted to 1:500 as the secondary antibodies. For CD8 and TIGIT multistaining, the antigen retrieval buffer was Tris-ethylenediaminetetraacetic acid (pH 9.0) at 98 °C. The primary anti- TIGIT antibody was mouse anti-TIGIT antibody (clone TG1, Dianova, Hamburg, Germany) diluted to 1:50. The secondary antibody was the same as above.

We examined the expression of the tumor markers PD-L1, and CD155 in paired samples obtained before and after preoperative therapy. For the TIL markers, PD-1 and TIGIT, the samples after preoperative therapy alone were evaluated because of the inability to evaluate tiny specimens before preoperative therapy with the above methods. Expression of PD-L1, CD155, PD-1, and TIGIT was independently evaluated by two of the investigators who were blinded to clinical data. PD-L1 and CD155 were recoded as tumor proportion scores (TPS). The expression rate of PD-1 or TIGIT was calculated as the ratio of CD8-positive and PD-1- or TIGIT-positive cells to CD8-positive cells. A discrepant result between two investigators was resolved by consensus on simultaneous examination.

### Statistical analysis

Proportions of categorical variables were compared by the chi-squared test or Fisher’s exact test, as appropriate. Continuous variables were compared using a Wilcoxon signed-rank test for paired data or the Mann–Whitney U-test for unpaired data. Receiver operating characteristic (ROC) curve analyses were done to decide the optimal cut-off value of the TPS and expression rate. The Kaplan–Meier method was used to assess the probability of OS, and survival differences were analyzed with the log-rank test. Differences were considered statistically significant when *p* values were < 0.05. All statistical analyses were performed with commercial software (SPSS version 27, IBM, Armonk, New York, USA).

### Ethics declarations

The institutional review board of the University of Occupational and Environmental Health, Japan approved the present study.

## Data Availability

The datasets used and analyzed during the current study are available from the corresponding author on reasonable request.
